# Managing the introduction of genomic applications into the National Health Service: A special challenge for health technology assessment in Italy

**DOI:** 10.3389/fpubh.2022.932093

**Published:** 2022-08-10

**Authors:** Erica Pitini, Giuseppe Migliara, Valentina Baccolini, Claudia Isonne, Elena Mazzalai, Federica Turatto, Carla Salerno, Federica Pagano, Maria Teresa Menzano, Corrado De Vito, Carolina Marzuillo, Paolo Villari

**Affiliations:** ^1^Department of Public Health and Infectious Diseases, Sapienza University of Rome, Rome, Italy; ^2^Italian Ministry of Health, General Directorate for Health Prevention, Rome, Italy

**Keywords:** health technology assessment (HTA), National Health Service, public health genomics, public health governance, Italy, narrative review, genomic test

## Abstract

In recent years, the rapid proliferation of genomic tests for use in clinical practice has prompted healthcare systems to use a health technology assessment (HTA) approach to distinguish valuable from unwarranted applications. In this study, we narratively review the Italian HTA mechanisms for medical devices (MDs), both at the national and regional levels, and discuss the opportunity and benefits of extending them to genomic technologies, for which a dedicated assessment path was advocated by the National Plan for Public Health Genomics in 2017. We found that the National Health Technology Assessment Program for MDs, completed in 2019, had developed a structured pathway for the HTA of MDs; it established a hub-and-spoke structure, run by a governmental institution, and put in place transparent methodological procedures to cover all four HTA phases (i.e., proposal and prioritization, assessment, appraisal, and dissemination). However, several factors have hindered its adoption, and the regions are at different stages of its implementation. For these reasons, efforts should be made to ensure its effective deployment, both at national and regional levels. In addition, we argue that to harmonize the institutional roles and methodological procedures adopted, the time has come to concentrate resources on a single pathway for the assessment of all technologies that include both MDs and genomic applications.

## Introduction

The past 20 years have witnessed remarkable growth in the genetic and genomic sciences. The discoveries of the Human Genome Project have permeated clinical practice and have fueled a new medical approach called precision medicine, where medical interventions are customized according to an individual's genome and specific environmental factors (1, 2). Consequently, public health genomics (PHG) has emerged as an attempt to translate genome-based knowledge and technologies into population health benefits responsibly and effectively (3, 4). Given the rapid proliferation of genomic tests for research, clinical practice, and also direct-to-consumer applications, one of the main concerns of PHG is to distinguish valuable applications from unwarranted interventions and to ensure that access to the former is as widely available as possible (5).

The formal procedure by which the value of particular health technology is determined is known as health technology assessment (HTA). According to a new and internationally accepted definition, HTA is a multidisciplinary process that uses explicit methods to consider the best available evidence for the dimensions of value of a health technology, which often includes clinical effectiveness, safety, cost and economic implications, ethical, social, and legal implications, and organizational aspects, as well as wider implications for the patient, their relatives, and the population (6). This process is particularly relevant in countries such as Italy, where health services are publicly funded, in that it informs decision-makers on how best to allocate the limited funds available for health interventions (7).

Italy has pioneered the development of PHG policies that aim to translate the results of genomic research into health practice; two national plans for PHG have been enacted to date, in 2013 and 2017 (8, 9). Concerning HTA, the most recent plan requires the development of a dedicated national HTA pathway for genomic technologies (10), although this has yet to be implemented. In contrast, HTA programs for medical devices (MDs) have been developed both at regional and national levels to ensure effective stewardship and to guarantee equal access to innovative technologies across the whole nation (11). In this study, we review the Italian HTA mechanism for MDs and discuss the opportunity and benefits of extending this mechanism to the assessment of genomic technologies instead of developing a dedicated path.

## Methods

The Italian healthcare system is decentralized, such that health governance is devolved to the 19 regions and two autonomous provinces (APs), hereafter collectively referred to as “the Regions.” The Regions are responsible for organizing and delivering health services that achieve the common health objectives decided by the central government. Accordingly, this review of existing HTA mechanisms for MDs was conducted both at the national and regional levels.

### National level

The search questions and the eligibility criteria for inclusion in the review were formulated to identify regulatory documents governing the process and methods for HTA of MDs at the national level. In December 2021, two researchers independently conducted a narrative review of the relevant pages of the websites of the main central healthcare institutions, namely, the Italian Ministry of Health and its supervised entities, i.e., the Italian Institute of Health (ISS), the National Agency for Regional Healthcare Services (AGENAS), and the Italian Medicine Agency (AIFA) (Supplementary Table S1). This search was supplemented by a free Google search with search terms in Italian that were appropriate to the topics of HTA and regulation. The relevant information was extracted independently using a standardized data abstraction form focused on three aspects: (I) HTA regulation (How is HTA of MDs regulated at a central level?); (II) HTA phases (Which HTA phases are specified at the central level? What methods are used in each phase?); and (III) Specifications for genomic applications (Are there any specifications for the HTA of genomic applications at the central level?). Any disagreement was resolved by consensus or discussed with a third researcher.

### Regional level

An official fact-finding survey on HTA activities in the Italian Regions, on behalf of AGENAS and the Italian HTA Society (SIHTA), was published in 2016 (12). The survey interviewed representatives of 17 regions (Campania, Friuli-Venezia Giulia, Molise, and Sardegna did not participate) to collect information on regional HTA processes and methods. Hence, as a first step, two researchers independently extracted the results of this survey for each region by adapting the same data abstraction form used at the national level to the regional level: (I) HTA regulation (How is HTA of MDs regulated at the regional level?); (II) HTA phases (Which HTA phases are specified at the regional level? What methods are used in each phase?); and (III) Specifications for genomic applications (Are there any specifications for the HTA of genomic applications at the regional level?). Second, the researchers updated these data through a narrative review aimed at identifying regulatory documents that described the process and methods for HTA of MDs at the regional level. In December 2021, the websites of the main regional healthcare institutions were queried (Supplementary Table S2), and a free Google search was performed using search terms in Italian appropriate to the topic of HTA regulation in each region. As the published survey was conducted at the end of 2015, only documents published since January 2016 were included; an exception was made for the four regions that did not participate in the original survey, for which no time limit was applied. Any disagreement was resolved by consensus or discussed with a third researcher.

## Results

The key features of the Italian HTA mechanism for MDs, both at the national and regional levels, are summarized in Table 1.

**Table 1:** Italian health technology assessment mechanism for medical devices: national and regional level.

**Level**	**HTA mechanism**	**Key features**
National Level	National HTA Program for MDs regulated by national ordinances	Structured pathway for the HTA of MDs run by a central governmental hub which networks with various spokes, including the Regions and other research and healthcare centers Standardized and evidence-based methodological procedures that cover all phases of the HTA (i.e., proposal and prioritization, assessment, appraisal, and dissemination)
Regional level	Regional HTA initiatives for MDs regulated by regional ordinances	Regional HTA pathways and methodologies appear to be heterogeneous About half the Regions seem to have formally acknowledged the NHTAPMD (11 Regions out of 21)

HTA, Health Technology Assessment; MDs, medical devices; NHTAPMD, National HTA Program for Medical Devices.

### HTA at the national level

We retrieved eight documents that set out the regulation of the process and methods for HTA of MDs at the national level, published between 2005 and 2021 (Table 2) (13–20).

**Table 2:** Retrieved central regulatory documents and stages of national HTA regulation.

**Stage**	**Document, year**	**Directives**
I. Need for a central HTA mechanism for MDs	National Health Plan 2006–2008, 2005 (13)	HTA is set as a national priority
	Health Pact 2014–2016, 2014 (14)	The need for an institutional framework for the HTA of MDs is recognized
II. Development of a central HTA mechanism for MDs	Stability Law for 2015, 2014 (15)	The MoH is commissioned to set up the NHTAPMD
	Stability Law for 2016, 2015 (16)	The NHTAPMD governance is entrusted to an inter-institutional SC composed of representatives of the MoH, AGENAS, AIFA and Regions
	MoH Decree on ISS regulation, 2016 (20)	The National center for HTA is established at ISS
	State-Regions Standing Conference PNHTADM Strategic document, 2017 (17)	The deal between the MoH and the Regions on the key elements of the NHTAPMD is established
III. Revision of the central HTA mechanism for MDs	Health Pact 2019–2021, 2019 (18)	To merge all functions fragmented between several institutions into a single entity that operates with the regional centers and oversees the governance of the entire HTA process
	European Delegation Law 2019–2020, 2021 (19)	A reorganization of the activities of institutions responsible for the governance of HTA of MDs is proposed, together with a strengthening of HTA functions on the basis of the objectives identified by the National HTA Program

AGENAS, National Agency for Regional Healthcare Services; AIFA, Italian Medicines Agency; HTA, Health Technology Assessment; ISS, Italian Institute of Health; MDs, medical devices; MoH, Ministry of Health; PNHTADM, National HTA Program for Medical Devices; SC, steering committee.

#### HTA regulation

Since the late 1990's, several diverse HTA initiatives have been implemented across Italy, both at the regional and local levels (21). The first attempt to coordinate HTA centrally can be found in a number of national health planning documents, which recognize HTA and the development of a central HTA mechanism for MDs as a national priority (Table 2—Stage I) (13, 14). Measures began to be put into practice with the 2015 and 2016 stability laws, which formally required the Ministry of Health to establish the National HTA Program for MDs (NHTAPMD), supervised by AGENAS and a steering committee (SC). The SC, composed of representatives of the Ministry of Health, AGENAS, AIFA, and the Regions, validates methodologies and coordinates the activities of the program (15, 16). The NHTAPMD was officially launched in 2017, with the main aim of establishing a collaborative network between the national and regional healthcare institutions for the HTA of MDs (Table 2—Stage II) (17). The process and methods of the program, described below, were developed by three main working groups and were completed in 2019. In the meantime, the Ministry of Health also endorsed the establishment of a National Center for HTA within the ISS, to perform HTA and encourage its use in the NHS (20). In 2019, within the State-Regions Standing Conference, the opportunity arose to merge all the functions previously fragmented between several institutions into a single entity entrusted with the governance of the entire HTA process. This idea anticipated the recent 2021 European Delegation Law, which advocated the reorganization and coordination of the activities of institutions responsible for the governance of HTA of MDs, together with a reinforcement of the NHTAPMD, including a permanent source of funding (Table 2—Stage III) (18, 19).

#### HTA phases

The NHTAPMD covers all the HTA phases, from the prioritization of technologies to be assessed (proposal and priority setting) to the collection of the scientific evidence (assessment), the final recommendation on adoption (appraisal), and their dissemination to institutions as appropriate (dissemination) (22).

##### Proposals and priority setting

Several stakeholders, i.e., healthcare institutions (national or regional), NHS facilities, and professionals; scientific societies; manufacturers; or patients and citizens (private or associations) may propose MDs for assessment through an *ad hoc* online system. Every 6 months, the SC prioritizes the proposed MDs for the further assessment using the following seven criteria: (1) impact on unmet healthcare needs; (2) ethical and social implications; (3) organizational impact; (4) economic and financial impact; (5) technical relevance; (6) clinical effectiveness; and (7) epidemiologic burden.

##### Assessment

The SC assigns the assessment of prioritized MDs to public or private collaborating centers (such as regions and academies) included in an *ad hoc* register or to technical governmental agencies (i.e., AGENAS or ISS), as appropriate. The assessment methodology relies on the EUnetHTA HTA Core Model, which is the European reference tool for the assessment of health technologies (23). Thus, the following nine evaluation dimensions are explored through the collection of scientific evidence: (a) health problems and current use of the technology; (b) description and technical characteristics of the technology; (c) safety; (d) clinical effectiveness; (e) cost and economic evaluation; (f) ethical analysis; (g) organizational aspects; (h) patient and social aspects; and (i) legal aspects.

##### Appraisal

This task is assumed by an *ad hoc* committee (appraisal committee; AC) composed of representatives from healthcare institutions (national and regional); NHS facilities; scientific research institutes and universities; scientific societies; and citizen and patient associations. The AC considers the scientific evidence summarized in the assessment report according to the following criteria: (i) healthcare need; (ii) added clinical value; (iii) sustainability; (iv) acceptability; (v) implementability; and (vi) feasibility. Then, it makes a judgment on whether the technology should be rejected, recommended, recommended for research purposes only, or recommended on the condition that additional real-world evidence on effectiveness and cost is generated. The SC revises the AC recommendations and decides whether to approve them. The final release is preceded by a 30-day public consultation period.

##### Dissemination and impact on decision making

The final results of the HTA process are published by the Ministry of Health and transmitted to the relevant national and regional institutions as appropriate to their expertise (22). In particular, a specific national commission is responsible for using the results of HTA to continuously update the so-called essential levels of care (LEA, i.e., the services that the NHS is required to provide to all citizens, free of charge or upon payment of a participation fee, with the public resources collected by general taxation) by excluding health interventions that have become obsolete and including innovative health interventions that, over time, prove to be effective for patient care (16, 17, 24). In turn, the Regions may use the results of the NHTAPMD to support those internal processes that aim to ensure the provision of the LEA (planning, purchasing, delivering, etc.) (17, 25).

#### Specifications for genomic applications

The NHTAPMD does not include any specific recommendation for the HTA of genomic applications.

### HTA at the regional level

**Figure 1:**
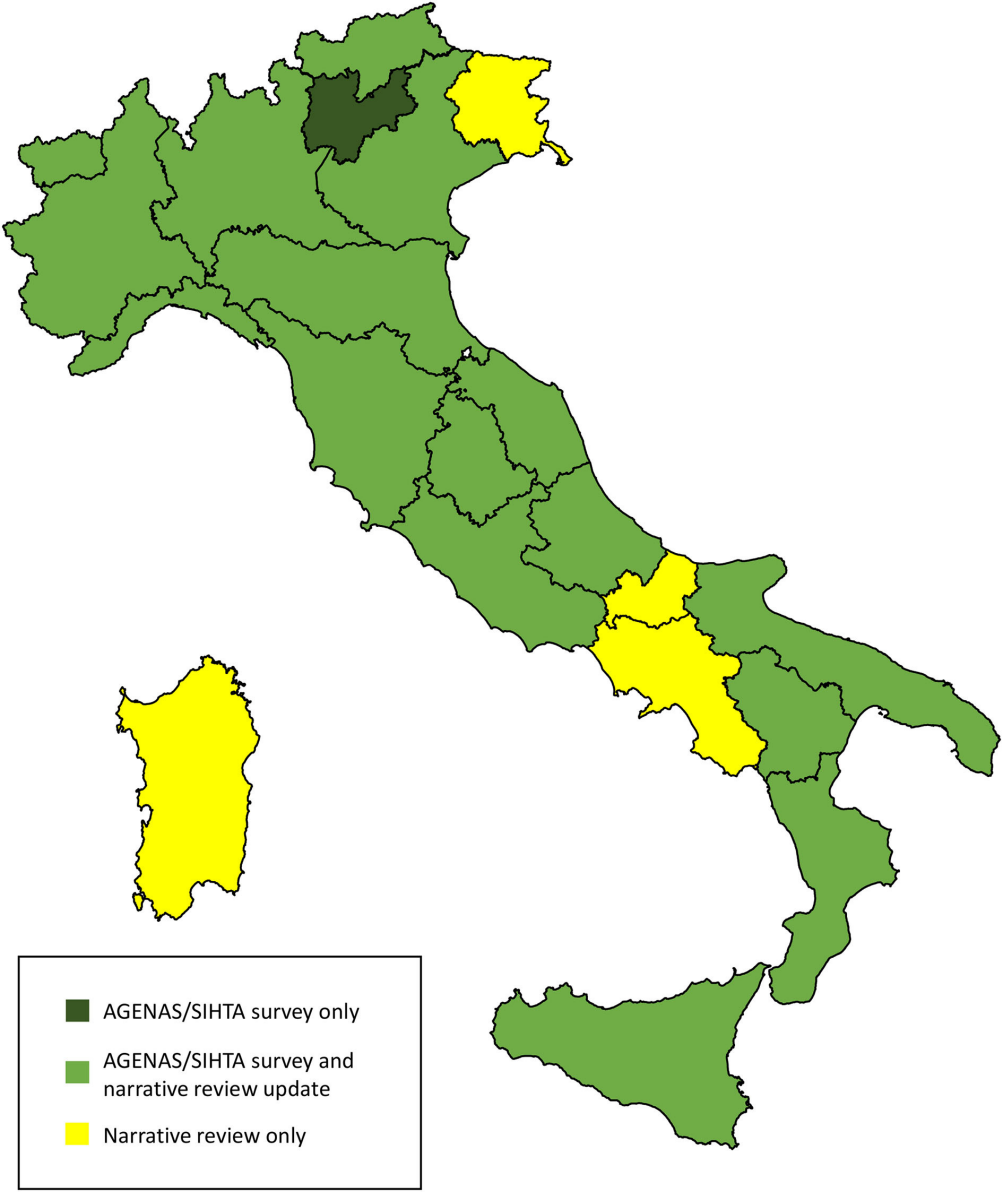
Source of information on the state of HTA in the Italian Regions. The map was adapted from the Italian National Institute of Statistics https://www.istat.it/storage/cartografia/confini_amministrativi/generalizzati/Limiti01012021_g.zip.

We found updated information on the state of HTA for 20 regions out of 21 (Figure 1). The retrieved regional regulatory documents are listed in Supplementary Table S3.

#### HTA regulation

Before or alongside the development of the national HTA strategy, almost all the Regions have ratified their own HTA initiatives into regional ordinances (20 regions out of 21, 95%; no regulation was found for Bolzano AP). Nevertheless, the level of development of such initiatives varies considerably, from regions that have only laid the groundwork for the establishment of an HTA program (e.g., Molise and Calabria) to regions where it is fully implemented (e.g., Lombardia and Emilia-Romagna). About half the Regions seem to have formally acknowledged the NHTAPMD (*n* = 11, 52%, i.e., Calabria, Emilia-Romagna, Lazio, Liguria, Lombardia, Marche, Puglia, Toscana, Trento AP, Umbria, and Veneto) and have incorporated it into their regulations, but only three appear as members of its Collaborating Centers Register (Veneto, Emilia-Romagna, and Puglia).

#### HTA phases

Regional HTA pathways appear to be heterogeneous. According to our findings, only about one-third of the Regions regulate all HTA phases (proposal and priority setting, assessment, appraisal, and dissemination), and in most cases, only fragmentary information was retrieved.

##### Proposals and priority setting

The stakeholders allowed to request that MDs be evaluated at a regional level include Regional Technical Committees, healthcare facilities and professionals, scientific societies, manufacturers, patients and citizens (private or associations), and universities. In the Regions where information was available, the prioritization was carried out by regional *ad hoc* committees. Prioritization criteria are not uniform across the Regions, but the most popular appears to be the organizational and economic impacts of the device.

##### Assessment

In the Regions where information was available, the assessment was performed by multidisciplinary regional teams, often including economists, epidemiologists, pharmacists, engineers, clinicians, and administrative staff. Regarding the assessment methodology, we found an explicit reference to the EUnetHTA HTA core model in about one-third of the Regions.

##### Appraisal

According to the available information, the appraisal was performed by an *ad hoc* regional committee that often included representatives from regional healthcare institutions and facilities, in addition to scientific experts on the topic of interest. Only a few regions clearly report the appraisal criteria and their application to multicriteria decision analysis methods (e.g., Sardegna and Lombardia); in most cases, information on appraisal methods was not available.

##### HTA dissemination and impact on decision making

At a regional level, the main decision-making processes that should be informed by HTA are the centralized purchasing of MDs and the planning and delivery of standardized healthcare pathways for the appropriate use of MDs, but these are not uniformly addressed in the retrieved documents (25). As mentioned above, other than by the HTA reports independently produced by the Regions, these decisions may also be informed by HTA reports produced within the NHTAPMD and made available to the Regions.

#### Specifications for genomic applications

No specific content for the HTA of genomics applications was found in the retrieved regional regulatory documents.

## Discussion

Due to the decentralized nature of the Italian healthcare system, several rather different regional initiatives for the HTA of MDs have emerged over the years and have been gradually regulated by regional ordinances. More recently, efforts were made to centralize the HTA of MDs at the national level while preserving the coordinated involvement of the Regions, culminating in the NHTAPMD. This program deployed a structured pathway for the HTA of MDs, which is run by a central governmental hub, where AGENAS has a leading role and networks with various spokes, including the Regions and other research and healthcare centers in the public register. Moreover, the NHTAPMD established standardized and evidence-based methodological procedures to cover all phases of the HTA, i.e., the proposal and prioritization of the MDs to be assessed; their assessment through evidence collection; and a final recommendation that aims at the adoption into the national healthcare system, with appropriate provision at the regional level. Unfortunately, full implementation of the NHTAPMD has been hampered by three factors, namely, the COVID-19 pandemic, as the definition of the program was completed in 2019; operational fragmentation and overlap between the central institutions involved in the program (i.e., Ministry of Health, ISS, AGENAS, AIFA); and a lack of agreement on financing mechanisms. Nevertheless, recent legislation has emphasized the need to improve the efficiency of the program by redefining the responsibilities and tasks of the central institutions involved and by involving manufacturers and distributors in its funding (18, 19). This may be particularly relevant in the current scenario, where the Regions are at different stages of adoption of the plan; such heterogeneity may contribute to increasing regional differences and may favor a return to past procedures, with disparate regional initiatives for the HTA of MDs.

Since genomic applications are MDs, it might be expected that their evaluation should be performed within the NHTAPMD. However, this opportunity needs to be examined in light of the specific requirements of the 2017 National Plan for PHG (10). This plan, in fact, called for the development of a separate HTA pathway for genomic technologies—centered on a different governmental hub, i.e., the ISS, with the role of research and healthcare spokes still to be defined—and for the adoption of dedicated procedures, with particular regard to the assessment methodology. Such a dedicated mechanism has not yet been instigated, perhaps because of the confusion it would cause regarding the institutional roles and procedures already established by the NHTAMD. Nevertheless, the National Plan for PHG is still in place and the dilemma over which route to take for the HTA of genomic applications needs a definitive answer.

The exceptional status of the HTA of genomic tests is not limited to Italy. As we have discussed elsewhere, several international entities have actively pursued dedicated pathways for genomic technologies, such as the UK Genetic Testing Network and the Australian Medical Service Advisory Committee, or have adopted *ad hoc* assessment methodologies, mostly a reflection of the well-known ACCE model (analytic validity, clinical validity, clinical utility, ethical legal, and social implications) developed in the United States (26–28). The special status reserved for genomic technologies could be partly explained by the expectations of the scientific community for their potential to drive the precision medicine revolution (29, 30). Additionally, questions have begun to arise about whether genomic technologies may pose particular challenges for HTA. Among the most-debated issues are the complexity of assessing the value of genomic information—specifically its potential to predict the onset or likelihood of disease, the possibility of unexpected findings or findings of unknown significance, and other implications that are not strictly health-related or that concern family or society more widely (31, 32). Another concern is the speed with which these new applications become available for the clinical practice: given that there might initially be few scientific evidence of their clinical benefits, a considerable commitment of resources is required to produce HTA reports in a reasonable time and to promote additional research to fill the evidence gaps (31).

Nevertheless, we believe that the challenges posed by genomic applications could be better addressed by taking advantage of the experience already developed for the HTA of other health technologies, particularly MDs. Instead of allocating resources to redundant mechanisms, efforts should be directed at considering whether the HTA pathways and methodologies already developed for MDs could be adjusted to meet the requirements of genomic applications without disrupting their overall structure. As for the specific Italian context, we believe that pursuing the development of a dedicated mechanism for genomic applications, as required by the National Plan for PHG, would create confusion regarding the institutional roles and methodological procedures established by the NHTAPMD. Moreover, the public nature of the Italian healthcare system, in which equity and resource constraints are a major concern, makes the use of common standards for the evaluation of all medical interventions the best way to help decision-makers identify those with the greatest health potential for the population, whether they are genomic-based or not.

The main limitations of our review are those deriving from the narrative approach, i.e., the non-reproducibility of the search, selection, and synthesis of the target documents, and a potential selection bias due to their availability on the web. This may apply especially to the regional search, as not all regions are equipped with an updated electronic repository. However, two researchers independently searched the institutional websites, meaning that all available information was likely to have been collected. In addition, the use of institutional sources should have ensured the objectivity of the retrieved information. Finally, as the search was run at the end of 2021, any document published in 2022 has not been included. Nevertheless, to the best of our knowledge, this is the first review that updates the 2016 SIHTA survey, providing timely evidence of the progress made.

## Conclusion

In this study, we have provided an updated overview of the HTA mechanisms in Italy. We collected information on the regulations and phases for HTA of MDs, and we found a discrepancy between the establishment of the national plan and its actual adoption throughout the country. For these reasons, more efforts should be made to ensure its full implementation both at the national and regional levels. In addition, the NHTAPMD could also serve as a basis for the assessment of genomic technologies. In fact, given the difficulties and challenges of implementing an effective national program, it could be the right time to concentrate resources on a single pathway for the assessment of all technologies instead of creating two separate routes, one for MDs and one to be used when the technology under assessment is a genomic application.

## Author contributions

EP, GM, VB, and PV contributed to the conception and design of the study. CI, EM, FT, CS, and FP performed the narrative review (bibliographic search and data extraction). EP and GM contributed to data curation. EP wrote the first draft of the manuscript. GM and VB wrote sections of the manuscript. PV, CM, CD, and MM contributed to supervision and funding acquisition. All authors contributed to manuscript revision and read and approved the submitted version.

## Funding

This study was supported by the Italian project Definizione e promozione di programmi per l'implementazione delle azioni centrali di supporto al Piano per l'innovazione del sistema sanitario basata sulle scienze omiche (Definition and promotion of programs to support the implementation of the Italian Omics science Plan), funded by the Italian Ministry of Health—National Center for Disease Prevention and Control.

## Conflict of interest

The authors declare that the research was conducted in the absence of any commercial or financial relationships that could be construed as a potential conflict of interest. The Handling Editor DT declared a past collaboration with the authors EP, GM, CD, CM, and PV.

## Publisher's note

All claims expressed in this article are solely those of the authors and do not necessarily represent those of their affiliated organizations, or those of the publisher, the editors and the reviewers. Any product that may be evaluated in this article, or claim that may be made by its manufacturer, is not guaranteed or endorsed by the publisher.
