# Supplementary material for: Managing the introduction of genomic applications into the National Health Service: A special challenge for health technology assessment in Italy
Source: Front Public Health. 2022 Aug 10;10:932093. doi: 10.3389/fpubh.2022.932093 (PMC9399489; doi:10.3389/fpubh.2022.932093)
Supplement: Supplementary file 1 [file Data_Sheet_1.docx]

Supplementary Material

# Supplementary Tables

Table S1 Institutional websites explored in the national narrative review

| INSTITUTION | WEBSITE |
| --- | --- |
| Italian Ministry of Health | https://www.salute.gov.it |
| Italian Institute of Health | https://www.iss.it |
| National Agency for Regional Healthcare Services | https://www.agenas.gov.it |
| Italian Medicines Agency | https://aifa.gov.it |

Table S2 Institutional websites explored in the regional narrative review

| REGION | WEBSITE |
| --- | --- |
| Abruzzo | https://www.regione.abruzzo.it  <https://sanita.regione.abruzzo.it>  http://www.asrabruzzo.it |
| Basilicata | https://www.regione.basilicata.it |
| PA Bolzano | <https://www.provincia.bz.it>  https://www.asdaa.it |
| Calabria | https://www.regione.calabria.it |
| Campania | http://regione.campania.it |
| Emilia Romagna | <https://www.regione.emilia-romagna.it>  https://salute.regione.emilia-romagna.it  https://assr.regione.emilia-romagna.it |
| Friuli Venezia Giulia | <https://www.regione.fvg.it>  https://arcs.sanita.fvg.it/it |
| Lazio | https://www.regione.lazio.it  https://www.salutelazio.it |
| Liguria | <https://www.regione.liguria.it>  https://www.alisa.liguria.it |
| Lombardia | https://www.regione.lombardia.it |
| Marche | <https://www.regione.marche.it>  https://www.asur.marche.it |
| Molise | https://www.regione.molise.it |
| Piemonte | http://www.regione.piemonte.it <https://www.sistemasalutepiemonte.it> |
| Puglia | <https://www.regione.puglia.it>  https://www.sanita.puglia.it |
| Sardegna | https://www.regione.sardegna.it  <https://www.sardegnasalute.it>  https://www.atssardegna.it |
| Sicilia | https://pti.regione.sicilia.it |
| Toscana | <https://www.regione.toscana.it>  https://www.ars.toscana.it |
| PA Trento | <https://www.provincia.tn.it>  https://www.trentinosalute.net |
| Umbria | https://www.regione.umbria.it |
| Valle d’Aosta | <https://www.regione.vda.it>  http://www.ausl.vda.it |
| Veneto | <https://www.regione.veneto.it>  https://salute.regione.veneto.it |

PA Autonomous Province

Table S3 Regional regulatory documents retrieved for the narrative review

| REGION | RETRIEVED DOCUMENTS |
| --- | --- |
| Abruzzo | DGR n. 53 del 28/03/2020. Dipartimento Sanità - Approvazione del nuovo assetto organizzativo e precisazione delle competenze e dei programmi da realizzare. (<https://www.regione.abruzzo.it/system/files/dgr/2020/DGR53_2020.pdf>) |
| Basilicata | Piano Regionale Socio-Sanitario 2018-2020. (https://www.aspbasilicata.it/sites/default/files/_PSR%202018-2020.pdf) |
| PA Bolzano | No documents found. |
| Calabria | DCA n. 57 del 26/02/2020. Approvazione Programma Operativo 2019-2021 predisposto ai sensi dell'art. 2 comma 88, della L. 23 dicembre 2009 n. 191 e s.m.i. (<https://www.regione.calabria.it/website/portaltemplates/view/view_provvedimenti.cfm?31424>) |
| Campania | DGR n. 586 del 21/11/2019. Presa d'atto e recepimento del "Piano Triennale 2019-2021 di sviluppo e riqualificazione del Servizio Sanitario Campano ex art. 2, comma 88, della Legge 23 dicembre 2009, n. 191", approvato con Decreto Commissariale n. 94 del 21/11/2019, ai fini dell'uscita dal commissariamento.(<http://burc.regione.campania.it/eBurcWeb/BurcPdfOutput/Burc_2019_11_26_12_26.pdf;jsessionid=6B926A70A756891CCD7C34A3DA58DE75.burcfe1>)  DGR n. 64 del 07/03/2011. Implementazione strumento innovativo gestionale HTA dell’*Horizon Scanning* nelle strutture sanitarie. (<http://burc.regione.campania.it/eBurcWeb/directServlet?DOCUMENT_ID=23368&ATTACH_ID=27933>) |
| Emilia-Romagna | DGR n. 2277 del 22/11/2019. Sistema regionale di governo dei dispositivi medici. Aggiornamento della DGR n. 1523 del 29 settembre 2008. (<https://servizissiir.regione.emiliaromagna.it/deliberegiunta/servlet/AdapterHTTP?action_name=ACTIONRICERCADELIBERE&operation=leggi&cod_protocollo=GPG/2019/2381&ENTE=1>)  DGR n. 977 del 18/06/2019. Linee di programmazione e di finanziamento delle Aziende e degli Enti del Servizio sanitario regionale per l’anno 2019. (<https://bur.regione.emilia-romagna.it/dettaglio-inserzione?i=a1d6eb59393f4173b8b3cb067019bd45>) |
| Friuli Venezia Giulia | Legge regionale n.27 del 17/12/ 2018. Assetto istituzionale e organizzativo del Servizio sanitario regionale. (<https://lexview-int.regione.fvg.it/FontiNormative/xml/scarico.aspx?ANN=2018&LEX=0027&tip=2&id=&lang=multi&a_ante=2019&n_ante=22&ci=1&vig=&idx=ctrl0&dataVig=19/12/2019>) |
| Lazio | DCA n. U00081 del 25/06/2020. Piano di riorganizzazione, riqualificazione e sviluppo del Servizio Sanitario Regionale 2019-2021. (<https://www.asl.rieti.it/fileadmin/user_upload/DCA_U00081_25_06_2020.pdf>)  DCA n. U00080 del 19/06/2020. Approvazione del Documento Programmatico sugli investimenti straordinari per l'ammodernamento.  strutturale, tecnologico ed organizzativo del Servizio Sanitario Regionale. Approvazione del Piano Decennale in Materia di Edilizia Sanitaria ex Art. 20, Legge 11 Marzo 1988, N. 67 Investimenti in Edilizia Sanitaria, Terza Fase – Stralcio 1B.1. (<https://anaao.lazio.it/index.php/dalla-regione-lazio/212-2020/345-giugno>)  DCA n. U00303 del 25/07/2019. Adozione del piano di rientro "Piano di riorganizzazione, riqualificazione e sviluppo del Servizio SanitarioRegionale 2019-2021" ai sensi e per gli effetti dell'art. 2, comma 88 della L. 191/2009, secondo periodo. (<https://www.asl.rieti.it/fileadmin/user_upload/Documenti/DCA/SAN_DCA_U00303_25_07_2019.pdf>)  DCA n. U00314 del 12/10/2016. Rettifica del DCA U00190 del 26/05/2016 avente ad oggetto: Piano Decennale in Materia di Edilizia Sanitaria ex Art. 20, Legge 11 Marzo 1988, N. 67 - Strutture Sanitarie - Approvazione del Programma Regionale di Investimenti in Edilizia Sanitaria, Terza Fase. (<https://www.anaao.lazio.it/index.php/files/74/SETTEMBRE-2016/654/101DCAU0031416.pdf>) |
| Liguria | DCS n. 495 del 30/12/2020. Disposizioni per l'organizzazione della rete ligure HTA e adozione del regolamento per il parere mini-Hta. (<https://www.alisa.liguria.it/components/com_publiccompetitions/includes/download.php?id=1349:deliberazione-del-commissario-straordinario-n-495-del-30122020-1349.pdf>)  DCS n. 43 del 20/02/2019. Ridefinizione del Gruppo di Coordinamento della Rete Regionale *Health Technology Assestment* (HTA) e aggiornamento delle modalità attuative. (<https://www.alisa.liguria.it/components/com_publiccompetitions/includes/download.php?id=1407:deliberazione-del-commissario-straordinario-n-43-del-20022019-1407.pdf>)  DCS n. 157 del 18/06/2018. Centri Collaborativi HTA nazionale ed attività / organizzazione della Rete Ligure HTA. (<https://www.alisa.liguria.it/components/com_publiccompetitions/includes/download.php?id=1939:deliberazione-del-commissario-straordinario-n-157-del-18062018-1939.pdf>)  DGR n. 531 del 10/06/2016. Disposizioni relative alla Rete regionale *Health Technology Assessment* (HTA). (<https://intranet.unige.it/sites/intranet.unige.it/files/DGR%20n%C2%B0531-2016%20-DISPOSIZIONI%20RELATIVE%20ALLA%20RETE%20REGIONALE%20HEALTH%20TECHNOLOGY%20ASSESSMENT%20(HTA)%20.rtf.pdf>) |
| Lombardia | DGR n. 2509 del 26/11/2019. Valutazione dell’appropriatezza d’uso di dispositivi biomedici e di tecnologie diagnostico-terapeutiche e riabilitative. Modifiche ed integrazioni alla DGR X/4831 del 22.02.2016 e alla DGR X/5671 del 11.10.2016. ([https://www.regione.lombardia.it/wps/wcm/connect/2ca0997a-d796-4db5-8649 6df4001c5292/Delibera+n.+2509+del+26+novembre+2019.pdf?MOD=AJPERES&CACHEID=ROOTWORKSPACE-2ca0997a-d796-4db5-8649-6df4001c5292-mWJvfNT](https://www.regione.lombardia.it/wps/wcm/connect/2ca0997a-d796-4db5-8649%206df4001c5292/Delibera+n.+2509+del+26+novembre+2019.pdf?MOD=AJPERES&CACHEID=ROOTWORKSPACE-2ca0997a-d796-4db5-8649-6df4001c5292-mWJvfNT))  Decreto n. 11776 del 16/11/2016 - Costituzione della Rete Regionale di Assessment HTA e delle Commissioni per le Tecnologie Sanitarie e adozione degli Schemi di riferimento operativi, ai sensi della DGR n. 5671 del 11/10/2016. (<https://www.regione.lombardia.it/wps/wcm/connect/5a0d2a18-2da4-47f3-b6d1-bf09dd471098/DDGW_11776_16.11.2016_HTA.pdf?MOD=AJPERES&CACHEID=5a0d2a18-2da4-47f3-b6d1-bf09dd471098>)  DGR n. 5671 del 11/10/2016. Ulteriori determinazioni e specificazioni in merito alla DGR x/4831/2016 “Nuove  determinazioni in merito alla valutazione dell’appropriatezza d’uso di dispositivi biomedici e di tecnologie diagnostico-terapeutiche e riabilitative”. (<https://www.regione.lombardia.it/wps/wcm/connect/83d4ea92-7686-45ec-bc1e-f04ad2d52d62/DGR_X_5671_11.10.2016.pdf?MOD=AJPERES&CACHEID=83d4ea92-7686-45ec-bc1e-f04ad2d52d62>)  DGR n. 4831del 22/02/2016 - Nuove determinazioni in merito alla valutazione dell’appropriatezza d’uso di dispositivi biomedici e di tecnologie diagnostico-terapeutiche e riabilitative. (<https://www.regione.lombardia.it/wps/wcm/connect/4c6c93cc-2eef-49d9-90f2-ad22f8d109cd/DGR_4831_2016_HTADM_RL.pdf?MOD=AJPERES&CACHEID=ROOTWORKSPACE-4c6c93cc-2eef-49d9-90f2-ad22f8d109cd-mnHXppD>) |
| Marche | DGR n.679 del 10/06/2019. Istituzione della Rete Regionale di *Health Technology Assessment* (HTA) della Regione Marche. (<http://www.norme.marche.it/Delibere/2019/DGR0679_19.pdf>)  DGR n. 427 del 15/04/2019. *Health Technology Assessment*: identificazione di una strategia e una metodologia per il disinvestimento di tecnologie non efficienti nel Servizio Sanitario Nazionale integrata tra il livello nazionale regionale e aziendale. Approvazione schema di Accordo di Collaborazione scientifica con l'Istituto Superiore di Sanità. (<http://www.norme.marche.it/Delibere/2019/DGR0427_19.pdf>) |
| Molise | DCA n. 94 del 09/09/2021. Programma operativo 2019-2021. Adozione. (<https://www.regione.molise.it/flex/cm/pages/ServeAttachment.php/L/IT/D/4%252Fc%252Fe%252FD.fe23ac4fcdf239450feb/P/BLOB%3AID%3D18186/E/pdf?mode=download>) |
| Piemonte | DGR n.82-5513 del 3/8/2017. Governo delle tecnologie biomediche e dell'innovazione in Sanità. Modifica D.G.R. n. 18-7208 del 10.3.2014. (<http://www.regione.piemonte.it/governo/bollettino/abbonati/2017/35/attach/dgr_05513_830_03082017.pdf>) |
| Puglia | DGR n. 1333 del 7/08/20. Intesa Stato Regioni Rep. Atti 157/CSR del 21 settembre 2017 concernente il Documento Strategico per il Programma Nazionale di *Health Tecnology Assessment* dei Dispositivi Medici (PNHTA DM). DGR 524/2018. Istituzione della Rete Pugliese HTA (RePuHTA). (<http://www.ager.puglia.it/documents/10192/53646611/DEL_1333_2020.pdf/92f2f94c-8934-4a3b-b504-2325d7671da4;jsessionid=EB5B47AABFCE0CD74167F899F592BCBF>)  Deliberazione del Direttore Generale AReSS Puglia n.185/2020. Atto di organizzazione e funzionamento del Centro Regionale HTA e della Rete Pugliese HTA. Sostituzione dell’atto di Regolamentazione attuativa del Centro Regionale *Health Technology Assessment* (CReHTA) ex DDG 210/2018. (<https://www.sanita.puglia.it/documents/45631926/45815728/Del.+185_2020+Atto+di+organizzazione+e+funzionamento+del+Centro+Regionale+HTA+e+della+Rete+Pugliese+HTA/52ff755c-7bbc-40c8-8a42-75b5a581f284>)  DGR n. 524 del 2018. Intesa Stato Regioni Rep.Atti 157/CSR del 21 settembre 2017 concernente il documento strategico per l’*Health Technology Assessment* (HTA). Recepimento. Individuazione Centro Regionale HTA. (<https://burp.regione.puglia.it/documents/20135/1155207/DEL_524_2018.pdf/c93fcacd-8345-3d38-ce0e-a9e258cac7b8?t=1622815037155>) |
| Sardegna | DCS n. 343 del 06/05/2021. Presa d’atto della Convenzione triennale tra l’Azienda per la tutela della salute – ATS Sardegna e l'Agenzia Nazionale per i Servizi Sanitari Regionali – AGENAS relativa ad attività di supporto, affiancamento e monitoraggio in ordine alle attività di cui alla L.R. n. 24 del 11 settembre. 2020 “Riforma del sistema sanitario regionale e riorganizzazione sistematica delle norme in materia. (<https://www.atssardegna.it/documenti/12_134_20210506175909.pdf>)  Legge regionale n. 24 del 11/09/2020. Riforma del sistema sanitario regionale e riorganizzazione sistematica delle norme in materia. Abrogazione della legge regionale n. 10 del 2006, della legge regionale n. 23 del 2014 e della legge regionale n. 17 del 2016 e di ulteriori norme di settore. (<https://buras.regione.sardegna.it/block/resource/LTc3ODU4NDc2OA==/inserzione.pdf>)  ATS Sardegna. Determinazione del Direttore del dipartimento di staff n. 3741 del 09/05/19. Costituzione rete per *assessment* HTA e identificazione componenti gruppo di lavoro per la valutazione delle tecnologie sanitarie ai sensi della Deliberazione ATS Sardegna n. 173 del 28/02/2019. (<https://www.atssardegna.it/documenti/12_134_20190509142208.pdf>)  ATS Sardegna. Deliberazione del Direttore Generale n. 173 del 28/02/2019. Linee di indirizzo per le attività di Assessment e modalità di funzionamento della Commissione per la valutazione delle Tecnologie Sanitarie nell’ATS Sardegna. (<https://www.atssardegna.it/documenti/12_111_20190228163540.pdf>) |
| Sicilia | DA n.1727 del 09/08/2019.Istituzione del Nucleo Tecnico Regionale per l'*Health Tecnology Assessment* (NTR-HTA). (<http://pti.regione.sicilia.it/portal/page/portal/PIR_PORTALE/PIR_LaStrutturaRegionale/PIR_AssessoratoSalute/PIR_Infoedocumenti/PIR_DecretiAssessratoSalute/PIR_DecretiAssessoriali/PIR_DecretiAssessorialianno2019/1727%2009.08.2019%20DA.pdf>) |
| Toscana | Decreto Dirgenziale n. 7468 del 17/05/2018. Commissione regionale di valutazione delle tecnologie ed investimenti sanitari. Costituzione Gruppo di Lavoro Regionale permanente sui Dispositivi Medici. (<http://www301.regione.toscana.it/bancadati/atti/Contenuto.xml?id=5181209&nomeFile=Decreto_n.7468_del_17-05-2018>)  DGR 1286 del 20/11/2017. DGR 302/2016: sviluppo del modello organizzativo del Sistema HTA regionale. Approvazione del regolamento interno del Centro Operativo ed approvazione delle procedure di valutazione e audizione. (<http://www301.regione.toscana.it/bancadati/atti/DettaglioAttiG.xml?codprat=2017DG00000001505>)  DGR n.302 del 11/04/2016. L.R. 40/2005, art. 10, comma 4 quinquies. Approvazione linee di indirizzo per le attività di valutazione e per le modalità di funzionamento della Commissione per la valutazione delle tecnologie e degli investimenti sanitari. (<http://www301.regione.toscana.it/bancadati/atti/DettaglioAttiG.xml?codprat=2016DG00000000380>) |
| PA Trento | Provincia autonoma di Trento, AGENAS. Relazione del progetto di Ricerca Autofinanziata “L’utilizzo di strumenti per il governo dei  dispositivi medici e per l’Health Technology Assessment (HTA) – (PRONHTA)”. Relazione 19 febbraio 2017- 15 maggio 2018. (<https://www.trentinosalute.net/Aree-tematiche/Innovazione-e-ricerca/Health-Technology-Assessment-HTA/Report/Relazione-del-progetto-di-Ricerca-Autofinanziata-L-utilizzo-di-strumenti-per-il-governo-dei-dispositivi-medici-per-l-Health-Technology-Assessment-HTA-PRONHTA>)  DGP n. 1915 del 16/11/17. Sistema provinciale dei controlli sulla qualità e sull'appropriatezza delle prestazioni sanitarie di ricovero e di specialistica ambulatoriale. Nomina del nucleo provinciale di controllo. (<http://www.delibere.provincia.tn.it/CercaSpecifica.asp?Modalita=Delibere&anno=2017&numero=1915>)  DGP n. 2412 del 20/12/2016. Costituzione del Programma TrentinoSalute 4.0 per la promozione e lo sviluppo dell'innovazione del Servizio Sanitario Provinciale e della sanità digitale. (<http://www.delibere.provincia.tn.it/CercaSpecifica.asp?Modalita=Delibere&anno=2016&numero=2412>) |
| Umbria | DGR n. 1439 del 04/12/2017. Istituzione del Centro regionale integrato per la Ricerca biomedica e dei servizi sanitari e per la Valutazione delle Tecnologie sanitarie (HTA) del Servizio Sanitario Regionale dell’Umbria (CRIVAT - Umbria). (https://www.regione.umbria.it/documents/18/12420709/DGR+1439+del+2017/98c693ad-f0fc-4091-9ee5-ca8af5531f9d)  DGR n. 58 del 25/01/2016. Istituzione della Struttura regionale per la Valutazione HTA (Nucleo di Valutazione regionale per *l’Health Technology Assessment* – Nucleo HTA) Determinazioni. (<http://docplayer.it/123693861-Cod-pratica-regione-umbria-giunta-regionale-deliberazione-della-giunta-regionale-n-58-del-25-01-2016.html>) |
| Valle d’Aosta | DGR n. 219 del 26/02/2018. Approvazione di indirizzi ed obiettivi di salute e di funzionamento dei servizi ed assegnazione del finanziamento all'azienda U.S.L. della Valle d'Aosta ai fini della definizione dell'accordo di programma 2018 e dell'adozione del bilancio preventivo economico annuale per l'anno 2018 e per l'anno 2019. Prenotazione di spesa. (<http://www.ausl.vda.it/elementi/www2016/areaospedaliera/1_delibera_copertina_testo_2018_219.pdf>) |
| Veneto | DGR n. 811 del 23/06/2020. Rinnovo della rete regionale per la governance dei dispositivi medici: istituzione del Tavolo tecnico regionale sui  dispositivi medici e attivazione delle Unità di valutazione aziendali delle richieste di acquisto di dispositivi medici. (<https://bur.regione.veneto.it/BurvServices/pubblica/html2pdf.aspx?id=422931&tipoAtto=9&storico=False>)  DGR n. 967 del 6/7/2018. Intesa tra il Governo le Regioni e le Province Autonome di Trento e Bolzano concernente il documento strategico per l'*Health Technology Assessement* dei dispositivi medici. Repertorio atti n.157/CSR del 21 settembre 2017. Recepimento. (<https://bur.regione.veneto.it/BurvServices/pubblica/html2pdf.aspx?id=373818&tipoAtto=9&storico=False>)  Legge Regionale 25 ottobre 2016, n.19 - Ente di *governance* della sanità regionale veneta denominato "Azienda per il governo della sanità della Regione del Veneto - Azienda Zero". Presa d'atto del trasferimento dei servizi tecnici per la valutazione dell'HTA (art. 2, comma 1 lett. g) punto 10). (<https://bur.regione.veneto.it/BurvServices/pubblica/html2pdf.aspx?id=332095&tipoAtto=11&storico=False>)  DRG n. 136 del 16/2/2016. Individuazione della struttura regionale titolare delle attività di valutazione delle tecnologie (*Health Technology Assessment*). Istituzione del Coordinamento Regionale *Health Technology Assessment* (CReHTA). Definizione funzioni e assegnazione compiti e finanziamento. (<https://bur.regione.veneto.it/BurvServices/pubblica/html2pdf.aspx?id=317657&tipoAtto=9&storico=False>) |
